# Predictors of Uncontrolled Periprosthetic Joint Infection Following Two-Stage Revision Total Knee Arthroplasty: An Extended Cohort Study

**DOI:** 10.3390/jcm15051875

**Published:** 2026-02-28

**Authors:** Chang-Jin Yon, Nae-Hwan Woo, Eun Seok Son, Ki-Cheor Bae

**Affiliations:** Department of Orthopedic Surgery, Keimyung University Dongsan Hospital, Keimyung University School of Medicine, Daegu 42601, Republic of Korea; poweryon@dsmc.or.kr (C.-J.Y.); 250271@dsmc.or.kr (N.-H.W.); esson@dsmc.or.kr (E.S.S.)

**Keywords:** total knee arthroplasty, periprosthetic joint infection, two-stage revision, risk factors, liver cirrhosis, sinus tract, fungal infection, erythrocyte sedimentation rate

## Abstract

**Background:** Two-stage revision arthroplasty is widely accepted as the reference standard for chronic periprosthetic joint infection (PJI) after total knee arthroplasty (TKA); however, reinfection or persistent infection occurs in a substantial subset of patients. We aimed to identify predictors of uncontrolled PJI following two-stage revision using an extended, single-center cohort. **Methods:** We retrospectively reviewed 177 knees with PJI after TKA treated with a uniform two-stage revision protocol between September 2011 and February 2022. Patients were classified as controlled (no further infection-related surgery or antimicrobial therapy ≥ 2 years after reimplantation) or uncontrolled (persistent infection after the first stage or reinfection after reimplantation). Demographics, comorbidities, laboratory parameters, perioperative factors, and microbiological characteristics were compared. Multivariable logistic regression with penalized estimation was used to identify independent predictors of uncontrolled infection. **Results:** Among 177 knees, 145 (81.9%) achieved infection control and 32 (18.1%) were classified as uncontrolled. On multivariable analysis using Firth penalized logistic regression, liver cirrhosis (odds ratio [OR] 9.87; 95% confidence interval [CI] 1.01–96.23; *p* = 0.049), the presence of a sinus tract at the first infection-control surgery (OR 3.47; 95% CI 1.43–8.40; *p* = 0.006), and fungal pathogens (OR 8.92; 95% CI 2.46–32.32; *p* = 0.001) were independently associated with uncontrolled PJI. Pre-reimplantation erythrocyte sedimentation rate (ESR) was significantly higher in the uncontrolled group on univariate analysis but was evaluated as a supportive marker due to limited availability in patients who did not undergo reimplantation. Demographic variables and most routine preoperative laboratory markers, including C-reactive protein before the first stage, were not independently associated with treatment failure. **Conclusions:** Liver cirrhosis, the presence of a sinus tract, and fungal infection are independent risk factors for uncontrolled PJI after two-stage revision TKA. Preoperative risk stratification incorporating host, local, and microbiological factors may assist in optimizing reimplantation timing, tailoring antimicrobial strategies, and improving patient counseling.

## 1. Introduction

Periprosthetic joint infection (PJI) remains one of the most serious complications following total knee arthroplasty (TKA), leading to substantial morbidity, increased healthcare costs, and a high risk of implant failure [1,2]. The incidence of PJI after primary TKA ranges from 0.5% to 2%, with even higher rates reported following revision procedures [3,4]. As the global volume of primary and revision TKA continues to increase, the absolute burden of PJI is expected to rise accordingly [5,6].

For chronic infections, two-stage revision arthroplasty is widely accepted as the reference standard, with reported infection eradication rates ranging from 70% to 90% [7,8,9]. This approach typically involves complete removal of all prosthetic components and cement, thorough debridement of infected and necrotic tissue, placement of an antibiotic-loaded cement spacer, pathogen-directed intravenous antimicrobial therapy, and subsequent reimplantation after an antibiotic-free interval [10,11]. Despite its widespread use and generally favorable outcomes, a substantial proportion of patients experience treatment failure, either as persistent infection following the first stage or as reinfection after reimplantation [12,13]. In the present study, these scenarios were collectively defined as “uncontrolled infection,” as both represent failure to achieve durable infection eradication and have comparable clinical consequences in terms of morbidity and need for additional intervention. Nevertheless, persistence after stage one and reinfection after reimplantation may arise from partially distinct biological mechanisms, a consideration that informed our subsequent analytical approach.

Accurately identifying patients at elevated risk of uncontrolled infection may refine clinical decision-making regarding the timing of reimplantation, the aggressiveness of debridement, the choice and duration of antimicrobial therapy, and patient counseling [14,15]. Previous studies have suggested multiple potential risk factors for failure after staged procedures, including host comorbidities, wound complications, serologic and inflammatory markers, and specific pathogens [16,17,18,19]. However, reported findings have been heterogeneous and frequently limited by modest sample sizes, variable definitions of treatment success or failure, and inconsistent surgical and antimicrobial protocols [20,21]. In addition, relatively few studies have evaluated host-related vulnerability, local tissue status, and microbiological characteristics simultaneously within a uniform institutional protocol, limiting the ability to derive stable and clinically actionable predictors.

Our institution previously reported outcomes from a cohort of 70 knees treated with a uniform two-stage protocol [22]. Building on that work, we analyzed an extended cohort of 177 knees managed over a longer study period using consistent surgical principles and standardized outcome definitions. By expanding the sample size while maintaining a uniform treatment framework, the present study aimed to reduce treatment-related heterogeneity and improve the stability of multivariable risk estimates. This study expands upon our previously published cohort [10] by incorporating additional cases and extended follow-up to further validate and refine predictors of uncontrolled infection.

The objective of this study was to identify independent predictors of uncontrolled PJI following two-stage revision TKA in an extended, single-center cohort treated with uniform protocols and prespecified outcome definitions.

## 2. Materials and Methods

### 2.1. Study Design and Ethical Approval

This retrospective cohort study was conducted at a tertiary academic center. The present study represents an extended cohort analysis, expanding upon a previously reported institutional series by including additional patients and an extended follow-up period. The study protocol was approved by the Institutional Review Board of Keimyung University Dongsan Medical Center (IRB approval number: 2025-11-025) and was conducted in accordance with the Declaration of Helsinki. The requirement for informed consent was waived due to the retrospective design, de-identification of data, and minimal risk to participants.

### 2.2. Setting and Surgical Protocol

All included cases were managed using a uniform and standardized two-stage revision protocol for chronic knee periprosthetic joint infection (PJI), consistent with established international guidelines [23,24].

Stage one consisted of complete removal of all prosthetic components and cement using hand instruments and high-speed burrs to ensure thorough cement extraction, followed by aggressive synovectomy and debridement of all infected, necrotic, or suspicious tissue. Copious pulsatile lavage was performed, and an antibiotic-loaded polymethylmethacrylate (PMMA) cement spacer was implanted. After removal of the components and cement, intramedullary canals of the femur and tibia were systematically debrided and irrigated. Multiple tissue samples (at least three to five) were obtained from different anatomical locations for microbiological analysis. An articulating antibiotic-loaded cement spacer was routinely used during the study period. Systemic intravenous antibiotics were administered according to culture results for a minimum of six weeks in collaboration with infectious disease specialists.

Intravenous pathogen-directed antibiotics were administered for approximately 6 weeks after stage one, guided by infectious disease consultation and culture sensitivities. After completion of intravenous antibiotic therapy, patients underwent an antibiotic-free interval of approximately 4 weeks, during which they were monitored clinically for signs of persistent or recurrent infection. Prior to reimplantation, all patients underwent comprehensive clinical evaluation, serologic testing (C-reactive protein [CRP] and erythrocyte sedimentation rate [ESR]), and joint aspiration for cell count, differential, and culture according to surgeon protocol [25].

Stage two involved spacer removal, repeat thorough debridement of all suspicious tissue, copious pulsatile lavage, and reimplantation using revision components. Metal augments and stems were utilized as indicated based on the severity of bone deficiency. When infection control was deemed adequate based on preoperative assessment, first-generation cephalosporins were administered for a short postoperative course (24–48 h). The timing of reimplantation was determined based on clinical assessment, normalization or downward trends in inflammatory markers, and absence of local signs of infection. At reimplantation, multiple intraoperative cultures were routinely obtained. Revision implants with stems and metal augments were used as indicated by the extent of bone loss. Postoperatively, patients received short-course prophylactic antibiotics when infection control was judged adequate, whereas extended antimicrobial therapy was considered if intraoperative cultures were positive.

**Figure 1:**
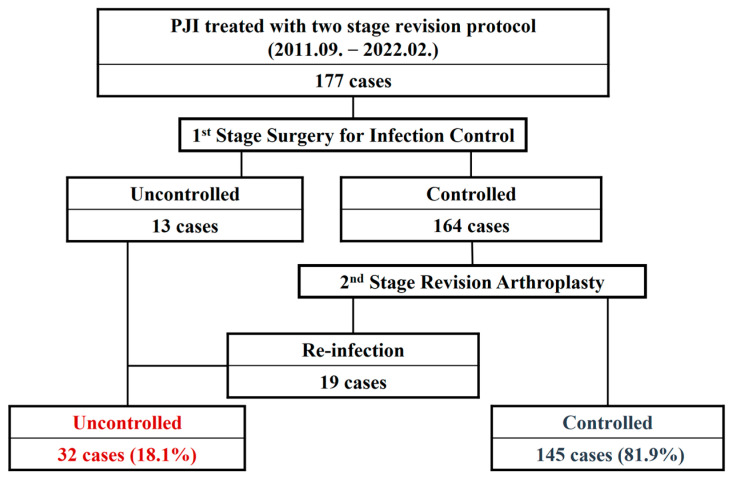
Flow diagram of patient selection and standardized two-stage revision protocol. Patients treated with two-stage revision for periprosthetic joint infection were included. Uncontrolled PJI was defined as persistent infection after stage one precluding reimplantation or reinfection after reimplantation requiring additional intervention.

A schematic flow diagram summarizing patient selection and the standardized two-stage revision protocol is shown in Figure 1.

### 2.3. Participants: Eligibility, Definitions, and Grouping

Inclusion criteria were: knees meeting accepted diagnostic criteria for PJI [26] following primary total knee arthroplasty (TKA); treatment with the standardized two-stage revision protocol between September 2011 and February 2022; and a minimum of 24 months of follow-up after reimplantation for patients who underwent stage two. Exclusion criteria included bilateral simultaneous cases, incomplete medical records, or less than 24 months of follow-up after reimplantation.

Outcome definitions were established a priori.

Controlled infection (Controlled group) was defined as the absence of additional infection-related surgery or antimicrobial therapy for at least 2 years after reimplantation, with resolution of clinical signs and symptoms of infection.

Uncontrolled infection (Uncontrolled group) was defined as persistent infection after stage one precluding safe reimplantation, or any clinically diagnosed reinfection after reimplantation requiring additional surgical intervention and/or prolonged antimicrobial therapy, including same-organism reinfection, different-organism reinfection, and culture-negative clinical infection. Both persistent infection after stage one and reinfection after reimplantation were categorized as uncontrolled infection because both represent failure to achieve durable infection eradication and are associated with comparable clinical consequences, including the need for further surgical intervention. We acknowledge that these scenarios may arise from partially distinct biological mechanisms; however, from a clinical outcome perspective, they were analyzed as a unified failure endpoint.

### 2.4. Variables and Data Collection

Data abstraction was performed using a predefined codebook to standardize variable definitions and minimize misclassification bias. Variables were selected based on clinical relevance and prior literature.

Collected variables included demographics (age, sex, body mass index [BMI], affected side, symptom intervals); comorbidities (tobacco use, cardiovascular disease, diabetes mellitus, cerebrovascular accident, dual antiplatelet therapy, immunocompromised status, liver cirrhosis, and mental health disorders); inflammatory and biochemical laboratory values (hemoglobin, albumin, estimated glomerular filtration rate [eGFR], CRP, and ESR) measured before stage one and before reimplantation; perioperative factors (blood transfusion, inflammatory arthritis, post-traumatic arthritis, presence of a sinus tract, prior joint infection, and bacteremia during the spacer period); and microbiological characteristics (pathogen categories including methicillin-sensitive organisms, methicillin-resistant organisms, Pseudomonas species, fungal infections, and polymicrobial infections). Immunocompromised status was defined as the presence of one or more of the following conditions: chronic corticosteroid use (≥5 mg/day prednisone equivalent for >3 months), active malignancy under treatment, solid organ transplantation, hematologic malignancy, human immunodeficiency virus infection, or other documented immunosuppressive therapy.

### 2.5. Microbiological Methods

For each surgical stage, multiple periprosthetic tissue specimens (minimum three to five) were collected under sterile conditions from distinct anatomical sites and sent for aerobic, anaerobic, and fungal cultures. Cultures were incubated for up to 14 days in accordance with institutional protocol. Antimicrobial selection and treatment duration were determined based on culture results in consultation with infectious disease specialists. Molecular diagnostic techniques such as polymerase chain reaction or routine sonication of explanted components were not routinely performed during the study period.

### 2.6. Statistical Analysis

Continuous variables were assessed for normality and summarized as appropriate. Variables with non-normal distributions are presented as median (interquartile range), whereas laboratory variables with approximately normal distributions are presented as mean ± standard deviation. Between-group comparisons were performed using the Mann–Whitney U test or independent *t*-test for continuous variables and the chi-square or Fisher’s exact test for categorical variables.

All clinically relevant variables were initially evaluated using univariate analyses. Variables demonstrating potential associations (*p* < 0.10) and those considered clinically important were subsequently entered into multivariable logistic regression models to identify independent predictors of uncontrolled infection. Results are reported as odds ratios (ORs) with 95% confidence intervals (CIs).

Given the limited number of uncontrolled events relative to the number of candidate predictors, particular attention was paid to the risk of model overfitting. The number of events per variable (EPV) was considered during model construction, and the final multivariable model was restricted to a limited number of clinically relevant predictors to preserve parsimony. To reduce potential small-sample bias, especially for sparse variables such as liver cirrhosis, Firth penalized logistic regression was applied as the primary multivariable approach. Conventional maximum likelihood estimates were also calculated for comparison.

Pre-reimplantation laboratory parameters, including ESR prior to revision TKA, were available only for patients who proceeded to reimplantation. Because a subset of uncontrolled cases represented persistent infection after stage one without reimplantation, inclusion of pre-reimplantation ESR in the primary multivariable model would have required exclusion of these clinically relevant failures. Therefore, the primary multivariable model was constructed using variables available for the entire cohort (N = 177), while pre-reimplantation ESR was evaluated separately in secondary analyses.

All analyses were performed using SAS software version 9.4 (SAS Institute Inc., Cary, NC, USA).

## 3. Results

### 3.1. Cohort Characteristics

A total of 177 knees in 177 patients were included in the final analysis, comprising 145 knees (81.9%) in the Controlled group and 32 knees (18.1%) in the Uncontrolled group. Baseline demographic and clinical characteristics of the study cohort are summarized in Table 1.

### 3.2. Univariate Analysis

All clinically relevant variables were initially assessed using univariate analyses. Comparisons between the Controlled and Uncontrolled groups are summarized in Table 2.

Among comorbidities, liver cirrhosis was significantly more frequent in the Uncontrolled group, while tobacco use was more frequent in the Uncontrolled group but did not reach statistical significance. Laboratory findings measured before stage one surgery did not differ significantly between groups; however, inflammatory markers assessed before reimplantation, particularly the erythrocyte sedimentation rate (ESR), were significantly higher in the Uncontrolled group.

With respect to perioperative and microbiological factors, the presence of a sinus tract at stage one surgery and fungal infection were both significantly more common in the Uncontrolled group. Complete univariate results are presented in Table 2. Among fungal infections, *Candida albicans* was the most frequently identified species, followed by non-albicans Candida species. Among methicillin-resistant organisms, methicillin-resistant *Staphylococcus aureus* and coagulase-negative staphylococci were predominant.

### 3.3. Multivariable Logistic Regression Analysis

After adjustment for potential confounders using Firth penalized logistic regression, three variables remained independently associated with uncontrolled infection. Liver cirrhosis, the presence of a sinus tract at stage one surgery, and fungal infection were identified as significant independent predictors of uncontrolled infection. Detailed results of the multivariable logistic regression analysis are shown in Table 3.

### 3.4. Treatment Outcomes

At a minimum follow-up of 2 years, the overall infection control rate was 81.9%. Among the 32 patients in the Uncontrolled group, 8 patients (25.0%) had persistent infection after stage one that precluded reimplantation, whereas 24 patients (75.0%) experienced reinfection after reimplantation.

## 4. Discussion

In this extended single-center cohort of 177 knees treated with a standardized two-stage revision protocol for periprosthetic joint infection (PJI), we identified three independent predictors of uncontrolled infection: liver cirrhosis, the presence of a sinus tract at the first infection-control surgery, and fungal pathogens. These predictors represent complementary dimensions of infection control failure, integrating host vulnerability, local disease burden, and pathogen-related characteristics, while residual systemic inflammation (e.g., pre-reimplantation ESR) may provide additional supportive information.

Among host-related factors, liver cirrhosis emerged as a strong predictor of uncontrolled infection, and the association remained significant after penalized regression analysis. Cirrhosis is well known to be associated with immune dysfunction involving impaired neutrophil activity, complement deficiency, and dysregulated cytokine responses, all of which may compromise the host’s ability to eradicate infection. In addition, altered pharmacokinetics and pharmacodynamics of antimicrobial agents in cirrhotic patients may further limit effective treatment. From a clinical perspective, these findings underscore the importance of recognizing liver cirrhosis as a high-risk condition when planning two-stage revision, warranting early multidisciplinary involvement and careful preoperative counseling regarding the likelihood of infection control [27,28,29,30,31]. In contrast, generalized immunocompromised status was not independently associated with treatment failure in our cohort. This finding suggests that specific host conditions such as liver cirrhosis may exert a more pronounced and clinically meaningful impact than broader immunosuppression categories.

Local disease burden also played a critical role in treatment failure. The presence of a sinus tract at the time of the first-stage surgery was independently associated with uncontrolled infection, reflecting chronic infection, compromised soft-tissue envelopes, and a direct conduit for microbial persistence. These findings suggest that surgeons should anticipate more extensive debridement in such cases and consider adjunctive strategies, including multidisciplinary assessment and, when indicated, staged soft-tissue procedures to optimize local conditions prior to reimplantation [32,33,34].

With respect to systemic inflammatory markers, ESR measured immediately prior to reimplantation was significantly higher in the Uncontrolled group on univariate analysis, whereas serologic markers obtained before stage one surgery did not differ meaningfully between groups. This finding suggests that residual inflammatory activity after initial source control may be more informative than baseline inflammatory burden at presentation. However, because pre-reimplantation ESR is available only in patients who undergo reimplantation, and because a subset of uncontrolled cases represent persistent infection after stage one without reimplantation, ESR was evaluated as a supportive marker rather than included in the primary multivariable model. Persistently elevated or non-declining ESR values may therefore serve as a warning signal, prompting caution in reimplantation timing and reinforcing the value of serial serologic monitoring during the interstage period [35,36,37,38,39,40].

Pathogen-related factors further influenced outcomes. Fungal infections were associated with a markedly increased risk of uncontrolled infection. Fungal PJI is inherently challenging due to diagnostic difficulties, low-grade inflammatory responses, biofilm formation, and limited antimicrobial options. Our findings are consistent with prior reports demonstrating poor outcomes following standard two-stage protocols in fungal PJI and support the need for tailored treatment strategies, including prolonged antifungal therapy and, in selected cases, consideration of alternative surgical approaches such as resection arthroplasty [41,42,43,44,45,46,47].

Overall, our results align with and extend existing literature on risk factors for failure after two-stage revision. Previous studies have highlighted the importance of host comorbidities and immunocompromised states as determinants of outcome, and our data further strengthen the evidence for liver cirrhosis as a specific high-risk condition [17,48,49].

The association between sinus tracts and treatment failure has also been reported in prior series [50,51]. In addition, while prior studies have reported inconsistent results regarding serologic markers, our observation that ESR measured immediately prior to reimplantation was higher in uncontrolled cases on univariate analysis suggests potential value as a supportive marker in selected patients [37,52]. The overall infection control rate observed in this cohort is comparable to success rates reported in recent systematic reviews, supporting the external validity of our findings [9].

This study has several strengths, including a relatively large single-center cohort managed with a uniform surgical and antimicrobial protocol, extended follow-up, and comprehensive assessment of host, laboratory, perioperative, and microbiological variables. Limitations include the retrospective design with potential residual confounding, single-center data that may limit generalizability, reliance on culture-based pathogen identification without routine use of molecular diagnostic techniques (e.g., sonication or polymerase chain reaction), which may have limited detection of low-grade biofilm-associated infection, and wide confidence intervals for some predictors reflecting low event rates. In addition, although liver cirrhosis emerged as a significant predictor, detailed stratification according to Child–Pugh class, MELD score, etiology of cirrhosis, and degree of hepatic decompensation was not consistently available due to the retrospective design and long study period. Therefore, residual heterogeneity among cirrhotic patients cannot be excluded. Despite these limitations, this study provides robust real-world evidence and offers clinically applicable insights for risk stratification in two-stage revision for PJI.

## 5. Conclusions

In this extended single-center cohort of patients undergoing two-stage revision for PJI after total knee arthroplasty, liver cirrhosis, the presence of a sinus tract at the first-stage surgery, and fungal pathogens were independently associated with uncontrolled infection. Pre-reimplantation ESR was significantly higher in the uncontrolled group on univariate analysis and may serve as a supportive marker of residual inflammatory activity. These factors provide a practical framework for preoperative risk stratification and clinical decision-making. Incorporating host, local, and microbiological risk factors into treatment planning may facilitate more judicious timing of reimplantation, optimization of antimicrobial strategies in high-risk patients, and realistic patient counseling. Prospective multicenter studies are warranted to validate these findings and to develop quantitative risk prediction models.

## Figures and Tables

**Table 1:** Baseline demographic and clinical characteristics of the study cohort.

	Controlled Group N = 145	Uncontrolled Group N = 32	*p* Value
	n (%) or Median (Q1, Q3)	n (%) or Median (Q1, Q3)	
Gender			0.7197 ^b^
Male	32 (22.1%)	8 (25.0%)	
Female	113 (77.9%)	24 (75.0%)	
Age (years)	69 (63, 72)	65 (60,72)	0.2816 ^a^
Affected Side (Rt.:Lt.)			0.4142 ^b^
Rt	75 (51.7%)	14 (43.7%)	
Lt	70 (48.3%)	18 (56.3%)	
Height (cm), Mean ± SD	156.3 ± 8.2	155.3 ± 7.5	0.5320 ^c^
Weight (kg), Mean ± SD	62.7 ± 9.6	64.5 ± 12.0	0.3581 ^c^
BMI	25 (23.2, 27.9)	26.8 (24.1, 29.1)	0.0881 ^a^
Diabetes Mellitus	57 (39.3%)	12 (37.5%)	1.0000 ^b^
Liver Cirrhosis	1 (0.7%)	3 (9.4%)	0.0212 ^b^
Immunocompromised Status	15 (10.3%)	3 (9.4%)	1.0000 ^b^
Interval between Symptom Onset and 1st infection control surgery for PJI (days)	74 (27, 182)	100 (19, 196)	0.8503 ^a^

^a^ Mann–Whitney U test; ^b^ Chi-square or Fisher’s exact test, as appropriate; ^c^ Independent Two-sample *t*-test.

**Table 2:** Univariate logistic regression analysis for factors associated with uncontrolled PJI (N = 177).

Variable	Controlled Group N = 145	Uncontrolled Group N = 32	OR (95% CI)	*p* Value
Host-related factors				
Tobacco use	4 (2.76%)	3 (9.38%)	3.64 (0.77–17.16)	0.1019
Cardiovascular disease	24 (16.55%)	4 (12.50%)	0.72 (0.23–2.24)	0.5713
Diabetes mellitus	57 (39.31%)	12 (37.50%)	0.93 (0.42–2.04)	0.8493
Stroke history	10 (6.90%)	1 (3.13%)	0.44 (0.05–3.53)	0.4362
Dual antiplatelet use	3 (2.07%)	1 (3.13%)	1.53 (0.15–15.18)	0.7174
Immunocompromised	15 (10.34%)	3 (9.38%)	0.90 (0.24–3.30)	0.8696
Liver cirrhosis	1 (0.69%)	3 (9.38%)	14.90 (1.50–148.29)	0.0212
Mental health disorder	5 (3.45%)	2 (6.25%)	1.87 (0.34–10.08)	0.4682
Pre-operative laboratory findings before 1st stage infection control surgery (mean ± SD)				
Hemoglobin, g/dL	11.2 ± 1.6	11.2 ± 1.3	1.03 (0.80–1.32)	0.8373
Albumin, g/dL	3.8 ± 0.4	3.9 ± 0.4	1.27 (0.49–3.26)	0.6241
eGFR, mL/min/1.73 m^2^	86.8 ± 30.4	88.6 ± 26.0	1.00 (0.90–1.02)	0.7560
CRP, mg/dL	7.7 ± 6.9	4.9 ± 3.4	0.95 (0.89–1.01)	0.1029
ESR, mm/h	87.0 ± 29.9	81.1 ± 29.3	0.99 (0.98–1.01)	0.3220
Pre-operative laboratory findings before 2nd stage revision TKA (mean ± SD)				
Hemoglobin, g/dL	11.6 ± 1.4	11.7 ± 1.4	1.02 (0.76–1.37)	0.8795
Albumin, g/dL	4.7 ± 0.6	4.0 ± 0.3	0.68 (0.24–1.90)	0.4600
eGFR, mL/min/1.73 m^2^	85.5 ± 26.5	86.9 ± 25.3	1.00 (0.99–1.02)	0.7929
CRP, mg/dL	0.5 ± 0.2	0.8 ± 0.4	1.46 (0.99–2.16)	0.0565
ESR, mm/h	39.0 ± 25.2	52.2 ± 26.1	1.02 (1.00–1.03)	0.0127
Peri-operative factors				
Peri-operative transfusion	105 (72.41%)	27 (84.38%)	2.06 (0.74–5.71)	0.1663
Sinus tract at 1st stage infection control surgery	21 (14.48%)	14 (43.75%)	4.54 (1.99–10.61)	0.0004
Inflammatory arthritis	2 (1.38%)	0 (0%)	0.88 (0.02–26.24)	0.9477
Post-traumatic arthritis	2 (1.38%)	1 (3.13%)	2.73 (0.02–37.12)	0.4057
History of joint infection before primary TKA	6 (4.14%)	2 (6.25%)	1.76 (0.30–8.03)	0.6046
Bacteremia at 1st stage	17 (11.72%)	0 (0%)	0.11 (0.01–2.09)	0.1430
Microbiological factors				
Methicillin-sensitive organisms	27 (18.62%)	3 (9.38%)	0.45 (0.12–1.59)	0.2169
Methicillin-resistant organisms	59 (40.69%)	8 (25.00%)	0.49 (0.20–1.16)	0.1024
Pseudomonas species	3 (2.07%)	2 (6.25%)	3.16 (0.51–19.71)	0.2189
Fungus	3 (2.07%)	8 (25.00%)	15.78 (3.91–63.70)	0.0001
Multiple pathogens	4 (2.76%)	3 (9.38%)	3.64 (0.77–17.16)	0.1019

Categorical variables are presented as n (%). Continuous variables are presented as mean ± SD. ORs are from univariate logistic regression.

**Table 3:** Multivariable analysis for independent predictors of uncontrolled PJI (Firth penalized logistic regression; N = 177).

Variable	Adjusted OR	95% CI	*p* Value
Liver cirrhosis	9.87	1.01–96.23	0.049
Sinus tract at stage one	3.47	1.43–8.40	0.006
Fungal infection	8.92	2.46–32.32	0.001

Firth penalized logistic regression was used to reduce small-sample bias due to sparse data (e.g., liver cirrhosis).

## Data Availability

The de-identified data presented in this study are available on request from the corresponding author. The data are not publicly available due to institutional review board restrictions and patient privacy considerations.
